# O.3.3-9 Working principles for intergenerational sports programs - scoping review

**DOI:** 10.1093/eurpub/ckad133.164

**Published:** 2023-09-11

**Authors:** Berry van Holland, Remo Mombarg, Saska Benedicic Tomat

**Affiliations:** Hanze University of Applied Sciences, The Netherlands; Hanze University of Applied Sciences, The Netherlands; ISCA, Denmark; b.j.van.holland@pl.hanze.nl

## Abstract

**Purpose:**

Generations Uniting through Sport (GUS) stimulates mutual understanding between youngsters and elderly people with sports activities. The project aims to promote intergenerational relations and increased physical activity by setting up an innovative and sustainable program. The concept for the GUS program is to identify, recruit, train and support a cohort of experienced practitioners. These professionals and volunteers will facilitate local-scale collaboration projects between youngsters and elderly people to increase their participation in physical activity and sport. The program will bring together young and old beneficiaries, through trained practitioners. The aim of the current research was to generate a state of the art overview of available evidence and practices that facilitate the development and implementation of intergenerational programs.

**Methods:**

The literature search consists of two parts. One part focuses on identifying scientific literature on working principles within intergenerational programs. The search will be performed in Google Scholar, Pubmed and PROSPERO and is restricted to literature from the year 2000 onwards and languages used within the consortium. Main search terms are ‘adolescent’, ‘elderly’, ‘intergenerational’, ‘activity’/’program’ and synonyms of these terms.

The second part of the search is performed within the project consortium and focuses on good practices from national and local settings. Searches will be performed in grey literature and through personal networks in France, Spain, Bulgaria, United Kingdom and Slovenia.

Literature analysis focuses on activities, target groups, involved stakeholders, behavioral change models, barriers and outcomes/impact.

**Results:**

The literature search results in a set of principles for programs and activities tailored towards generations uniting through sport/movement in Europe. More specific, it synthesizes behavioral change models incorporating methodologies that motivate youngsters and elderly people to exercise together and reduces some of the motivational and structural barriers to intergenerational programs.

**Conclusions:**

The final results and conclusions will be presented at the conference.

